# Supplementary material for: Thromboinflammation Model-on-A-Chip by Whole Blood Microfluidics on Fixed Human Endothelium
Source: Diagnostics (Basel). 2021 Jan 29;11(2):203. doi: 10.3390/diagnostics11020203 (PMC7911484; doi:10.3390/diagnostics11020203)
Supplement: Supplementary file 1 [file diagnostics-11-00203-s001.pdf]

Thromboinflammation Model-On-a-Chip by Whole Blood Microfluidics on Fixed Human Endothelium

Alexander Dupuy [1], Lejla Hagimola [1], Neil S.A. Mgaieth [1], Callum B. Houlahan [1], Renee E. Preketes-Tardiani [1], Paul R. Coleman [1,2] and Freda H. Passam [1,*]

Supplementary Information

Methods

1. Modification of Endothelialized Chip for the Study of vWF in Thromboinflammation.

Microfluidic channels were coated with fibronectin (100 μg/mL) and then incubated overnight at 4°C. HUVECs were cultured to 90% confluency. Cells were detached from culture flasks using Trypsin/EDTA and resuspended at 10×10^6 cells/mL and 10 μL was perfused through the microfluidic channel. Cells were left to settle and spread on the fibronectin channel for 1 h, at 37 °C, 5% CO2, then stimulated with or without TNF-α (10 ng/ml) for 4 h, with regular media changes every hour. HUVEC biochips were then perfused with media containing TNF-α (10 ng/mL) at shear rate $1000s^{-1}$ for 10 min. Biochips were then fixed with paraformaldehyde (4% w/v) and stained with Alexa Fluor 594 conjugated anti-vWF antibody (2 μg/mL), anti-ICAM-1-APC antibody (2 μg/mL) for 30 min, Hoechst (3 μg/mL) for 15 min at room temeperature, and imaged by confocal microscopy.

Results

Application of shear (1000 s-1) to TNF-α treated live HUVECs resulted in increased secretion of vWF and the formation of vWF strings (**Supplementary Figure S1**). This modified chip can be used for the study of endothelial vWF in thromboinflammation.

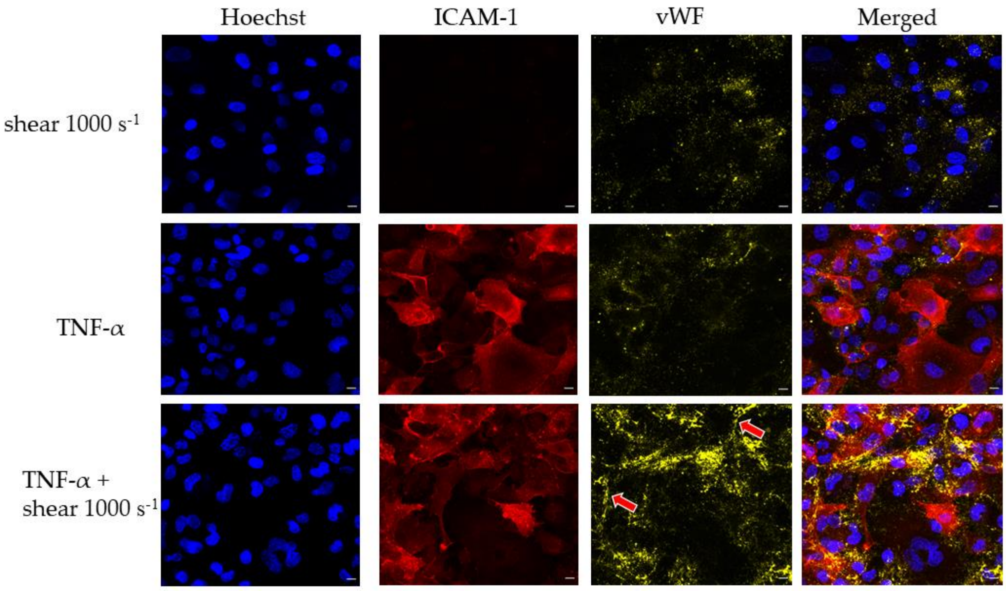

Supplementary Figure S1. Treatment of live HUVECs with TNF-α and shear promotes vWF secretion and vWF string formation. Expression of surface adhesion proteins on live HUVECs, coated on the chip, with 3 different treatments: perfusion of media at 1000 s-1 for 10 mi ("shear 1000 s-1), TNF-α treatment 10 ng/ml for 4 h ("TNF-α"), TNF-α treatment 10 ng/ml for 4 h followed by perfusion of media at 1000 s-1 for 10 min ("TNF-α + shear 1000 s-1"), then fixed, then stained. Confocal micrographs showing maximum intensity projections of z-stacks stained for nuclei (blue), ICAM-1 (red), and vWF (yellow). Red arrows denote the presence of vWF strings. Scale bar represents 10 μm.
